# Assessing the Efficacy of Ortho GPT: A Comparative Study with Medical Students and General LLMs on Orthopedic Examination Questions

**DOI:** 10.3390/bioengineering12121290

**Published:** 2025-11-24

**Authors:** Philippe Fabian Pohlmann, Maximilian Glienke, Richard Sandkamp, Christian Gratzke, Hagen Schmal, Dominik Stephan Schoeb, Andreas Fuchs

**Affiliations:** 1 Department of Urology, Medical Center-University of Freiburg, Faculty of Medicine, University of Freiburg, 79106 Freiburg, Germany; 2 Department of Orthopedics and Trauma Surgery, Medical Center-University of Freiburg, Faculty of Medicine, University of Freiburg, 79106 Freiburg, Germany; 3 Department of Orthopedic Surgery, University Hospital Odense, 5000 Odense, Denmark; 4 MVZ Sportorthopädie Freiburg, 79110 Freiburg, Germany

**Keywords:** Artificial Intelligence, medical education, orthopedics, natural language processing, student assessment, machine learning

## Abstract

Background: Domain-specific large language models (LLMs) like Ortho GPT have potential advantages over general-purpose models in medical education, offering improved factual accuracy and contextual relevance. This study evaluates the performance of Ortho GPT against general LLMs and senior medical students on validated orthopedic examination questions. Methods: Six LLMs (Ortho GPT 4o, ChatGPT 4o, ChatGPT 3.5, Perplexity AI, DeepSeek-R1, and Llama 3.3-70B) were tested using multiple-choice items from final-year medical student orthopedic exams in German language. Each model answered identical questions under standardized zero-shot conditions; accuracy rates and item-level results were compared using McNemar’s test, Jaccard similarity, and point-biserial correlation with student difficulty ratings. Results: Ortho GPT achieved the highest accuracy across models. McNemar’s tests revealed the significant superiority of Ortho GPT over DeepSeek (*p* = 2.33 × 10^−35^), Llama 3.3-70B (*p* = 1.11 × 10^−32^), and Perplexity (*p* = 4.01 × 10^−5^). Differences between Ortho GPT and ChatGPT 4o were non-significant (*p* = 0.065), suggesting near-equivalent performance to the strongest general model. No LLM showed correlation with student item difficulty (|r| < 0.07, *p* > 0.05), indicating that models solved items independently of human-perceived difficulty. Jaccard indices suggested moderate overlap between Ortho GPT and ChatGPT 4o, but distinct response profiles compared with general LLMs. Conclusions: These findings illustrate the superiority of Ortho GPT in orthopedic exam accuracy and context relevance, attributed to its specialized training data. The domain-specific approach enables performance matching or exceeding top general LLMs in orthopedics, emphasizing the importance of domain specialization for reliable, curriculum-aligned support in medical education.

## 1. Introduction

In recent years, large language models (LLMs) such as ChatGPT, GPT-4, and Llama have rapidly evolved from general conversational systems into powerful tools capable of processing, reasoning, and generating complex textual information [1]. Their accessibility and versatility have sparked a wave of experimentation across education, research, and healthcare. In medicine, LLMs are increasingly used to summarize clinical knowledge, draft documentation, support diagnostic reasoning, and serve as interactive learning companions. Early studies have shown that general-purpose models can achieve passing scores on standardized medical licensing examinations such as the USMLE or national board exams, suggesting that such systems can approximate foundational medical knowledge [2,3].

However, these models remain limited by their generalist nature [4,5,6]. Their training data, though vast, are not optimized for the precision, terminology, and conceptual integration required in clinical reasoning. As a result, general LLMs may produce superficially plausible but inaccurate or inconsistent medical statements—a phenomenon that poses significant challenges for educational and clinical reliability [7]. This gap has led to growing interest in domain-specific LLMs—systems fine-tuned on curated datasets representing a distinct area of medical expertise. By aligning the model’s linguistic and conceptual structures with a specialty’s knowledge base, domain-specific fine-tuning can enhance factual accuracy, reduce hallucination rates, and improve contextual reasoning [7,8].

The critical research gap our work addresses lies in evaluating whether a specialty-focused LLM, meticulously fine-tuned on curated, high-quality resources in orthopedics, can outperform both established general LLMs and domain-external specialist models on standardized exam formats [9]. Prior initiatives have not systematically compared the cognitive and educational impact of truly domain-specific LLMs, nor have they quantified potential advantages for learner support in an objective, exam-based setting. Our approach extends beyond descriptive comparison by directly measuring differences in accuracy, error profile, and contextual reasoning on item-level performance [10].

Within medical education, such models have the potential to become transformative learning tools [11,12]. They can provide tailored explanations, generate specialty-specific exam content, simulate case discussions, and offer immediate, adaptive feedback aligned with curricular objectives [13]. Importantly, domain specialization may also support cognitive alignment—helping learners internalize reasoning patterns that mirror expert approaches within their field [14].

Orthopedics provides a particularly fitting test case for this concept. The discipline combines biomechanical understanding, imaging interpretation, and procedural decision-making, all of which demand structured domain knowledge that generic models often lack. Ortho GPT is a domain-specific AI model engineered for orthopedic medicine, designed to integrate academic literature, textbooks, and validated question banks for superior specialty reasoning. Unlike generic LLMs, Ortho GPT leverages orthopedic-focused data to enhance clinical accuracy and contextual depth in diagnostic reasoning and knowledge assessment. It is accessible for educational and research use via yeschat.ai or through the ChatGPT surface, targeting tasks such as clinical case simulation, exam preparation, and decision-support tailored explicitly to the needs of orthopedic learners and practitioners [15,16,17].

Based on these considerations, our study sets out to systematically compare Ortho GPT, general-purpose LLMs, and senior medical students on standardized orthopedic examination questions, explicitly formulating the following research questions:

- Does a domain-specific LLM (Ortho GPT) outperform generic and broadly biomedical LLMs in accuracy and contextual reasoning in orthopedics?
- How does its performance correlate with human difficulty ratings and established exam benchmarks?
- What evidence supports the use of such a model as an educational aid for orthopedic training?

Our findings offer quantitative and qualitative evidence regarding the value of domain specialization in LLMs and provide a new reference point for further work in specialty-specific AI-supported medical education [18,19].

The remainder of this paper is organized as follows: Section 2 details the materials and methods, including question selection, model evaluation, and statistical analyses. Section 3 presents the results, covering overall performance, item-level comparisons, and similarity analysis. Section 4 discusses key findings, limitations, and implications for medical education. Section 5 offers conclusions and directions for future research.

## 2. Materials and Methods

### 2.1. Study Design

This study employed a quantitative, comparative design to evaluate the performance of a domain-specific large language model (Ortho GPT) in answering orthopedic examination questions. The model’s accuracy and response patterns were compared with those of several general-purpose LLMs and a cohort of senior medical students. The study aimed to determine whether fine-tuning on domain-specific data leads to measurable improvements in accuracy and contextual relevance within medical education.

### 2.2. Question Dataset

The evaluation dataset consisted of 207 multiple-choice questions (MCQs) drawn from seven validated orthopedic examinations administered in the final year of medical studies, with each exam typically comprising 30 questions (Type A, Type A-, and Pick N formats). Each item was answered by approximately 180 students, resulting in a total pool of around 1240 individual examinees. All questions followed the single-best-answer or corresponding variant format and were verified by domain experts. This allowed us to robustly correlate item difficulty, based on anonymized student performance data, with model outcomes across a comprehensive range of orthopedic subtopics.

### 2.3. Models Evaluated

Six large language models were included in the comparison:

- ChatGPT 3.5 (OpenAI, 2023)
- ChatGPT 4o (OpenAI, 2024)
- Perplexity AI (Perplexity Inc., 2024)
- DeepSeek-R1 (DeepSeek AI, 2024)
- Llama 3.3-70B (Meta AI, 2024)
- Ortho GPT, a domain-specific model fine-tuned on orthopedic literature, clinical guidelines, and question banks.

### 2.4. Procedure

Each model was queried using identical prompts under standardized zero-shot conditions, meaning no prior examples, hints, or feedback were provided. Questions were presented one by one, and the full text of each question and response options was included in the prompt. The models’ outputs were recorded and mapped to one of the available multiple-choice options.

The temperature parameter was not modified in our experiments. All model responses were generated using the default temperature settings of each language model, with masks reset before every run to ensure a fresh context [20].

To minimize confounding effects, no model was provided with explanations or additional context, ensuring comparability across systems. Human student data were obtained retrospectively from the same question set under supervised exam conditions. Data collection was performed in August 2025.

#### Evaluation Metrics

The primary outcome measure was accuracy, defined as the proportion of correctly answered questions per model.

Secondary analyses were conducted to provide a deeper understanding of model behavior beyond overall accuracy and to increase transparency around our statistical choices.

- The McNemar test was used for pairwise comparisons between models on a question-by-question basis. As a non-parametric test for paired nominal data, it is ideal for determining whether one model significantly outperforms another on the same set of binary outcomes—in this case, correct versus incorrect responses. This approach accounts for the dependent structure of matched items and avoids the limitations of unpaired statistical comparisons.
- To assess the similarity of response patterns, we calculated the Jaccard similarity index, which measures the degree of overlap in correctly answered items between two models. This index complements accuracy scores by capturing whether models are solving the same questions correctly, thereby providing insight into shared or divergent strengths. A high Jaccard value implies similar item-level performance, while a lower value suggests unique or complementary capabilities.
- Finally, the point-biserial correlation coefficient was computed to assess the relationship between model correctness (binary: correct vs. incorrect) and item difficulty, defined as the proportion of students who answered each question correctly. This analysis was applied to determine whether model performance was systematically associated with human-perceived item difficulty.

### 2.5. Statistical Analysis

All analyses were conducted at the item level. McNemar tests were applied for pairwise model comparisons, with significance defined as *p* < 0.05 after Bonferroni correction for multiple testing.

Correlation analyses were performed using Pearson’s point-biserial coefficient (r_pb), with corresponding two-tailed *p*-values. Effect sizes were interpreted according to Cohen’s conventions.

Data visualization (bar plots, correlation scatter plots, and heatmaps for model overlap) was performed using Python (v3.11) and the statsmodels and matplotlib libraries.

### 2.6. Ethical Considerations

The study used anonymized, de-identified educational data without patient information. Ethical approval was therefore not required under institutional policy. The analysis adhered to standards for responsible AI research and reproducibility in medical education.

## 3. Results

### 3.1. Overall Accuracy

**Figure 1:**
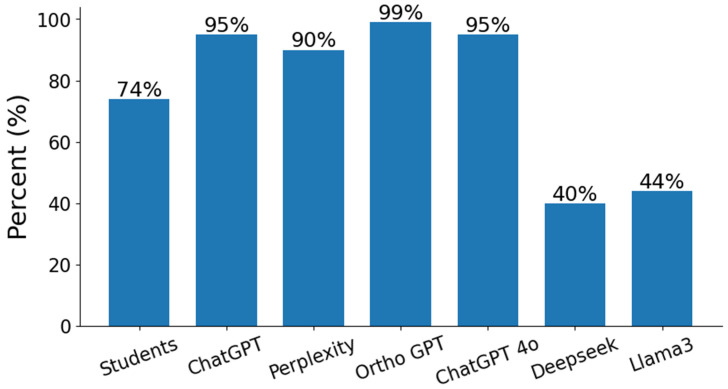
Overall accuracy (%) achieved by each large language model on exam items, with Ortho GPT demonstrating the highest proportion of correct responses.

The analysis explored whether large language models (LLMs) performed differentially on exam items based on their difficulty level for medical students. Point-biserial correlation coefficients revealed negligible associations between item difficulty and model accuracy for all tested models (Ortho GPT: r = −0.004, *p* = 0.959; ChatGPT: r = −0.064, *p* = 0.365), as depicted in Figure 1. Ortho GPT achieved the highest overall accuracy across all models, but no model demonstrated a systematic advantage on easier versus more difficult questions. This suggests that LLM performance is consistent across varying levels of item difficulty, indicating that the superior accuracy of Ortho GPT is not attributable to preferential success with questions of a specific difficulty level. Figure 1 thus contextualizes these findings within the broader analysis and demonstrates that exam item difficulty does not mediate LLM accuracy in this assessment (Figure 1).

### 3.2. Item-Level Analyses

**Figure 2:**
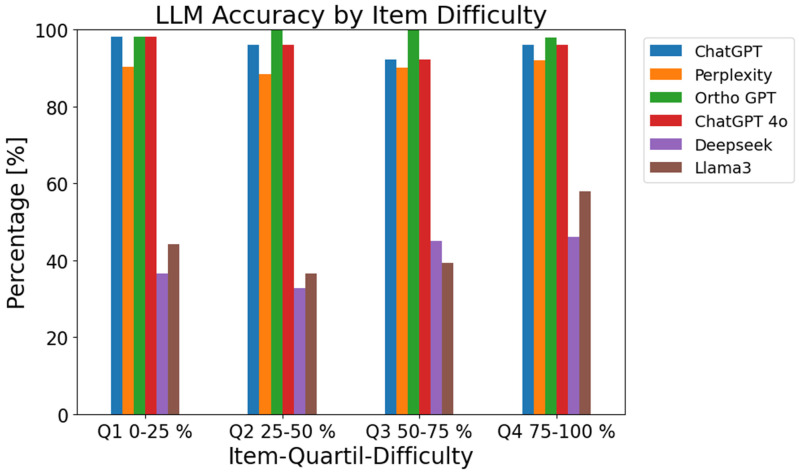
Accuracy of large language models (LLMs) across item difficulty quartiles. Difficulty was determined based on the proportion of correct answers given by final-year medical students. Items were grouped into quartiles from most difficult (Q1: 0–25% correct) to easiest (Q4: 75–100% correct).

None of the tested models exhibited a meaningful or significant correlation with student performance, indicating that LLM responses are independent of how easy or difficult individual items were for human examinees (Figure 2).

Accordingly, item difficulty for medical students does not explain the variance in LLM accuracy. In practical terms, LLMs do not mirror human response patterns and are not biased toward items that are more challenging or easier for students—there is no evidence that the models systematically outperform on questions where humans struggle or vice versa, as detailed in Table 1.

### 3.3. Comparative Accuracy Between LLMs: McNemar Test

To compare LLMs directly on their performance for individual exam questions, McNemar tests were applied. These tests reveal whether one model is significantly more likely to answer the same item correctly compared to another. Several pairwise comparisons showed extremely significant differences between models (Table 2).

These *p*-values are much lower than 0.05, indicating that some models—most notably Ortho GPT—consistently outperform others on the same set of orthopedic exam questions. However, some comparisons (e.g., ChatGPT vs. Ortho GPT, *p* = 0.0654) did not reach significance, suggesting similarity in performance between certain models.

### 3.4. Effect Sizes for McNemar Comparisons: Cohen’s g

To supplement the interpretation of statistical significance, Cohen’s *g* was calculated for each pairwise comparison. This effect size quantifies the magnitude of model differences, with values above 0.8 typically considered large. All significant comparisons demonstrated very large effects (Cohen’s *g* > 9), further supporting the superior accuracy of the leading models (notably Ortho GPT) beyond mere statistical significance. Non-significant comparisons generally yielded lower or uninterpretable effect sizes (NaN) Table 3.

### 3.5. Similarity of Response Patterns

**Figure 3:**
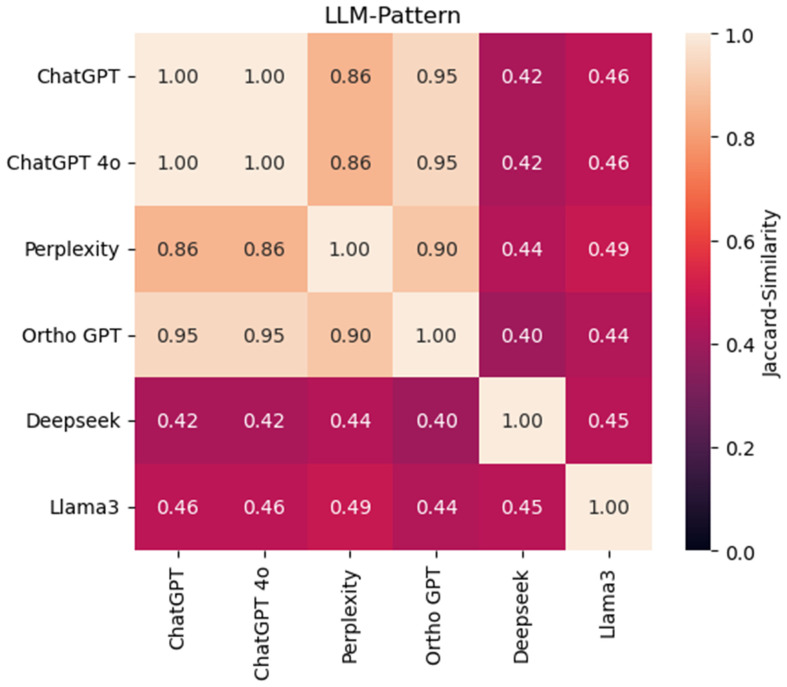
Jaccard similarity values (with 95% confidence intervals) indicate moderate overlap in correctly answered items between Ortho GPT and ChatGPT 4o.

Figure 3 illustrates the Jaccard similarity values for the correct responses between large language models, specifically highlighting the overlap between Ortho GPT and ChatGPT 4o. The Jaccard index, ranging from 0 (no overlap) to 1 (perfect overlap), quantifies how similarly two models answer the same items correctly. The moderate similarity observed in Figure 2 indicates that while the models share some strengths—correctly solving overlapping sets of questions—they also exhibit unique response patterns and make distinct errors. This analysis provides insights beyond overall accuracy, helping to characterize not just the commonalities in model behavior but also the complementary or divergent aspects of their performance across specific content areas (Figure 3).

### 3.6. Interpretation

All Overall, Ortho GPT—fine-tuned specifically for orthopedics—demonstrates marked advantages in accuracy and item-level performance compared to general-purpose LLMs like Deepseek and Llama3. The lack of correlation between human and model accuracy implies that LLMs approach questions differently from students. These findings emphasize the educational value of targeted training: specialty-specific models like Ortho GPT can provide more reliable and context-appropriate support, moving beyond what generalized AI can offer. This supports integrating domain-specific LLMs in medical curricula and assessments for improved educational outcomes.

## 4. Discussion

The findings of this study highlight the educational potential of domain-specific language models in medicine [7,21]. Ortho GPT consistently demonstrated higher accuracy than non-specialized LLMs, underscoring the importance of domain adaptation for factual precision and contextual reasoning [22]. This advantage can be attributed to the model’s exposure to orthopedic textbooks, guidelines, surgical case descriptions, and terminology-rich literature, which strengthen its semantic understanding of the discipline. In contrast, general LLMs—though powerful in general reasoning—lack the fine-grained conceptual mappings required for clinical subspecialties [8].

Despite the differences in training scope and domain specificity, Ortho GPT and ChatGPT-4o achieved comparable performance on orthopedic exam questions in this study. This result can be attributed to the architectural advancements and general reasoning capabilities of ChatGPT-4o, which allow it to generalize effectively across diverse topics. However, Ortho GPT’s specialized training on orthopedic literature, guidelines, and case data provides enhanced contextual precision and domain-relevant reasoning, especially for tasks requiring subspecialty knowledge [23]. The near-equivalence in quantitative outcomes underscores that general LLMs with sufficient complexity can approach specialist model accuracy, but domain adaptation remains crucial for context-appropriate support, reliability, and educational alignment in clinical training [24].

Beyond raw performance, Ortho GPT’s contextual relevance and lower rate of misleading reasoning are particularly noteworthy for medical education [12]. In clinical disciplines such as orthopedics, where diagnostic interpretation often depends on biomechanical logic and pattern-based differentiation, contextual correctness is as critical as answer accuracy. The ability of Ortho GPT to provide coherent justifications for its answers may therefore enhance learners’ conceptual understanding and offer a foundation for explainable AI-assisted education [25].

The absence of correlation between human item difficulty and model accuracy further illustrates a central difference between algorithmic and human cognition. LLMs appear to solve items through linguistic and associative reasoning rather than conceptual problem-solving [26]. This detachment from human error patterns may represent both an advantage—unbiased consistency—and a limitation—lack of pedagogical empathy. Future educational integration should therefore focus on hybrid systems that blend AI-generated explanations with instructor-guided reflection to foster metacognitive learning rather than rote answer reproduction [13].

From an educational standpoint, domain-specific LLMs like Ortho GPT can play several complementary roles. They may serve as adaptive tutors, capable of generating tailored case discussions, rationales, and formative assessments aligned with curricular standards. In assessment design, such models can support question validation by identifying ambiguities or unintended cues. In continuing medical education, they could assist clinicians in maintaining up-to-date knowledge in rapidly evolving subfields, reducing cognitive load through context-filtered summarization [27,28].

### 4.1. Error Analysis

Typical LLM errors in orthopedics and in general included misdiagnosed cases, omission of key management steps, anatomical inaccuracies, and “hallucinated” plausible-sounding but incorrect statements. These findings stress the importance of expert review and clear guidelines for instructional use [28,29].

### 4.2. Limitations

This study has several limitations that should be acknowledged. First, the question set—while representative of German orthopedic examinations—was limited in size, scope, and geographic relevance. Extending such analyses to broader datasets covering surgical decision-making, imaging, interprofessional reasoning, and exam formats from other countries would be necessary to generalize the findings across varied clinical settings and educational systems [26,27].

Second, the evaluation focused solely on multiple-choice accuracy, which does not fully capture the educational value, reasoning transparency, or interactive abilities of large language models [30].

Third, because all exam questions and student responses were in German, the possibility of linguistic bias must be considered. Language-specific phrasing, terminology, and cultural conventions may limit generalizability to other languages or multilingual scenarios in medical education—this is especially important for global applications [31].

Fourth, although Ortho GPT was fine-tuned on orthopedic data, the underlying model architecture and corpus remain proprietary, precluding full transparency regarding data provenance and possible biases. As with all LLMs, there remains a risk of “hallucinated” or confidently inaccurate answers [32].

Finally, the study used static, zero-shot prompts; performance may differ in more authentic, conversational, multimodal, or few-shot contexts [33].

### 4.3. Ethical Considerations, Curricular Integration and Safety Considerations

Widespread integration of AI language models into clinical education also raises important ethical and safety considerations. It is essential that LLM-assisted learning be supervised by qualified instructors who can detect inaccuracies, address algorithmic bias, and ensure the responsible use of model outputs [22].

Bias mitigation strategies should include regular updates, validation across diverse datasets, and transparent reporting of data provenance where possible. Clinical education programs incorporating domain-specific LLMs must provide guidance on appropriate use, highlight the potential for errors or “hallucinations,” and train learners to critically evaluate AI-generated information [25].

Responsible adoption requires ongoing evaluation of both accuracy and fairness, with clear boundaries set to avoid unsupervised clinical application and to safeguard patient welfare.

To advance beyond the current theoretical comparison, future research should include a case study or experimental intervention assessing Ortho GPT’s direct impact on learning outcomes or clinical comprehension. For example, a classroom trial with pre- and post-intervention assessments could measure actual improvements in medical student performance following model-assisted instruction.

## 5. Conclusions

This study shows that domain-specific large language models, such as Ortho GPT, can match or surpass general-purpose LLMs in answering medical examination questions while providing more contextually precise and clinically aligned reasoning. Fine-tuning on orthopedic literature and terminology appears to improve factual accuracy, interpretive consistency, and educational relevance.

Beyond raw performance, Ortho GPT exemplifies how specialization enables AI systems to reflect expert reasoning patterns rather than relying on generic associations. Such models can serve as adaptive learning partners—offering explanations, generating exam-style questions, and supporting self-directed study—provided they are used under appropriate supervision.

The findings underline that the future of AI in medical education lies in collaborative intelligence: a synergy between expert oversight and domain-optimized LLMs. When transparently trained and responsibly integrated, specialized models like Ortho GPT have the potential to enhance both the efficiency and depth of medical learning across disciplines.

## Figures and Tables

**Table 1:** None of the models showed correlation with students’ perceived difficulty.

Model	r	*p*-Value
ChatGPT	−0.064	0.365
Perplexity	0.027	0.705
Ortho GPT	−0.004	0.959
ChatGPT 4o	−0.064	0.365
Deepseek-R1	0.032	0.653
Llama 3.3-70B	−0.053	0.452

**Table 2:** McNemar’s test confirms superiority of Ortho GPT. *** p < 0.001.

Model	*p*-Value	Sig
Ortho GPT vs. Deepseek	2.33 × 10^−35^	***
ChatGPT vs. Deepseek	9.63 × 10^−35^	***
ChatGPT 4o vs. Deepseek	9.63 × 10^−35^	***
Ortho GPT vs. Llama3	1.11 × 10^−32^	***
ChatGPT vs. Llama3	4.93 × 10^−32^	***
ChatGPT 4o vs. Llama3	4.93 × 10^−32^	***
Perplexity vs. Deepseek	1.97 × 10^−31^	***
Perplexity vs. Llama3	1.01 × 10^−28^	***
Perplexity vs. Ortho GPT	4.01 × 10^−5^	***
ChatGPT vs. Perplexity	6.14 × 10^−2^	
Perplexity vs. ChatGPT 4o	6.14 × 10^−2^	
ChatGPT vs. Ortho GPT	6.54 × 10^−2^	
Deepseek vs. Llama3	3.21 × 10^−1^	
ChatGPT vs. ChatGPT 4o	1.00 × 10^0^	

**Table 3:** Pairwise McNemar test *p*-values and effect sizes (Cohen’s g) for model comparisons on orthopedic exam questions. Statistically significant differences (*p* < 0.05) and effect sizes (*g* > 0.8) indicate substantial disparities in accuracy between mode. *** p < 0.001.

Comparison	*p*-Value	Sig	Cohen’s *g*	Interpretation
Ortho GPT vs. Deepseek	2.33 × 10^−35^	***	10.910	Large effect
ChatGPT vs. Deepseek	9.63 × 10^−35^	***	10.677	Large effect
Perplexity vs. Ortho GPT	4.01 × 10^−5^	***	4.025	Large effect
ChatGPT vs. Perplexity	6.14 × 10^−2^		2.043	Large effect
ChatGPT vs. Ortho GPT	6.54 × 10^−2^		2.111	Large effect
ChatGPT vs. ChatGPT 4o	1.00 × 10^0^		NaN	No effect

## Data Availability

Data supporting the findings of this study are available from the corresponding author upon reasonable request. The data are not publicly available due to local data protection regulations.

## Abbreviations

The following abbreviations are used in this manuscript:

AI	Artificial Intelligence
LLM	Large Language Models
MCQ	Multiple-Choice Question
USMLE	United States Medical Licensing Examination
